# Endovascular Aneurysm Sealing: Sealed, Signed, Disposed?

**DOI:** 10.1016/j.ejvsvf.2025.05.006

**Published:** 2025-05-23

**Authors:** Martin Teraa, Joost A. van Herwaarden

**Affiliations:** Department of Vascular Surgery, University Medical Centre Utrecht, Utrecht, the Netherlands

In their recent study, Berge and colleagues compare the long term follow up outcomes of the Nellix endovascular aneurysm sealing system (EVAS, *n* = 117) with endovascular aneurysm repair (EVAR, *n* = 164) for elective aorto-iliac aneurysm treatment based on a single centre retrospective study.^1^ Both patient groups were treated between January 2013 and December 2016. Technical success was 100% for both treatment options. All cause mortality was not significantly different at 30 days, one year, or at long term (median 9.3 years, interquartile range 9.1, 9.5 years) follow up. The incidence of type II endoleaks was significantly lower in EVAS (2.6%) than in EVAR (32.2%, *p* < 0.001). However, EVAS was related to a higher device failure rate (46% *vs*. 2% at five years, *p* < 0.001), risk of ≥5 mm aneurysm sac growth during follow up (47% *vs*. 17.1%, *p* < 0.001), and related hereto open surgical conversion (OSC) was performed more often in EVAS (35.6% *vs*. 2.4%, *p* < 0.001). Eventually, aneurysm related mortality was significantly higher in the EVAS group (12.0% *vs*. 0.6%, *p* < 0.001).

After the introduction of the Nellix EVAS in 2011 it was received with great enthusiasm. With the conceptual change of filling the aneurysm sac with a biostable polymer filled endobag to attain fixation and apposition to the aneurysm sac's wall it was felt that Nellix would address the Achilles heels of EVAR, i.e., device migration and (type II) endoleaks. Additionally, it also had the potential to treat more challenging short irregular and conical necks. However, midterm results questioned the promising early results of EVAS with reports of device migration and endobag separation leading to type Ia endoleak, sac pressurisation and expansion. These device failures often occur beyond two years of follow up and pooled estimated incidence of type I endoleak, device migration, and re-intervention are all in the range of 25% after a mean follow up of two years.^2^ These worrying figures urged for a focused update of the 2019 ESVS Abdominal Aortic Aneurysm Clinical Practice Guidelines on how to deal with patients treated with the Nellix device.^3^ Ultimately, the manufacturer of the Nellix (Endologix, Inc., Irvine, CA, USA) stopped its production in 2022.

Despite the promising short term results of EVAS, also supported by the current study of Berge and colleagues, and the tempting concept, it failed in the long term. However, the disappearance of EVAS left us with the remaining Achilles heel of EVAR, i.e. endoleaks and related re-interventions. The concept that active aneurysm sac management could be beneficial is supported by the fact that sac shrinkage after EVAR is correlated with better outcomes. Aneurysm sac shrinkage after EVAR of at least 5 mm is associated with lower risk of death, secondary re-intervention, late aneurysm related complications, and risk of rupture compared with those patients with a stable or growing aneurysm sac.^4^ The paradigm of active aneurysm sac management is supported by evidence that pre-emptive embolisation of aortic side branches in conjunction with EVAR reduces the incidence of type 2 endoleaks, aneurysm sac growth, and re-interventions.^5^ However, pre-emptive side branch embolisation is associated with additional procedure time^6^ and radiation exposure.^7^ A novel alternative building on this concept of active aneurysm sac management and influencing sac behaviour led to the introduction of Impede-FX RapidFill by Shape Memory Medical Inc. (San Jose, CA, USA). Impede-FX RapidFill is a porous, radiolucent, embolic scaffold that is crimped to enable catheter delivery and expands on contact with blood. The shape memory polymer is delivered in the aneurysm sac during EVAR to fill the aneurysm surrounding a regular endograft to promote aneurysm thrombosis and sac shrinkage.^8^ It is currently being investigated in the pivotal Abdominal Aortic Aneurysm Sac Healing and Prevention of Expansion (AAA-SHAPE) trial (clinicaltrials.gov; NCT06029660). AAA-SHAPE will randomise 180 patients (2:1 ratio) with an infrarenal AAA to either regular EVAR combined with sac management with Impede-FX RapidFill or regular EVAR. Key outcomes will be sac diameter and volume change, rates of endoleaks, and re-interventions.

Although long term mortality in the study of Berge and colleagues did not differ between EVAS and EVAR, the high rates of device failure underlines the relevance of recommendation 123 of the ESVS 2024 Clinical Practice Guidelines on the Management of Abdominal Aorto-Iliac Artery Aneurysms.^9^ This recommendation states that use of novel techniques and concepts, including EVAS, is not recommended in routine clinical practice and should be confined to clinical studies until properly evaluated. And even after initial success long term structured and systematic surveillance of novel devices is mandatory to guarantee effectiveness and safety for our patients.
